# E-health literacy and associated factors among chronic patients in a low-income country: a cross-sectional survey

**DOI:** 10.1186/s12911-020-01202-1

**Published:** 2020-08-06

**Authors:** Kirubel Biruk Shiferaw, Binyam Chakilu Tilahun, Berhanu Fikadie Endehabtu, Monika Knudsen Gullslett, Shegaw Anagaw Mengiste

**Affiliations:** 1 Health Informatics Department, Medicine and Health science college, Debre Markos University, Debre Markos, Ethiopia; 2 Health Informatics Department, College of Medicine and Health science, University of Gondar, Gondar, Ethiopia; 3 Faculty of Health & Social Sciences, Science center Health & Technology, University of South-Eastern Norway, Notodden, Norway; 4 School of Business, Institute of Business, History & Social Sciences, University of South-Eastern Norway, Notodden, Norway

**Keywords:** eHealth literacy, Chronic patients, eHealth, Low income country, Electronic health

## Abstract

**Background:**

Chronic patients persistently seek for health information on the internet for medication information seeking, nutrition, disease management, information regarding disease preventive actions and so on. Consumers ability to search, find, appraise and use health information from the internet is known as eHealth literacy skill. eHealth literacy is a congregate set of six basic skills (traditional literacy, health literacy, information literacy, scientific literacy, media literacy and computer literacy). The aim of this study was to assess eHealth literacy level and associated factors among internet user chronic patients in North-west Ethiopia.

**Methods:**

Institutional based cross-sectional study design was conducted. Stratified sampling technique was used to select 423 study participants among chronic patients. The eHealth literacy scale (eHEALS) was used for data collection. The eHEALS is a validated eight-item Likert scaled questionnaire used to asses self-reported capability of eHealth consumers to find, appraise, and use health related information from the internet to solve health problems. Statistical Package for Social science version 20 was used for data entry and further analysis. Multivariable logistic regression was used to examine the association between the eHealth literacy skill and associated factors. Significance was obtained at 95% CI and *p* < 0.05.

**Result:**

In total, 423 study subjects were approached and included in the study from February to May, 2019. The response rate to the survey was 95.3%. The majority of respondents 268 (66.3%) were males and mean age was 35.58 ± 14.8 years. The multivariable logistic regression model indicated that participants with higher education (at least having the diploma) are more likely to possess high eHealth literacy skill with Adjusted Odds Ratio (AOR): 3.48, 95% CI (1.54, 7.87). similarly, being government employee AOR: 1.71, 95% CI (1.11, 2.68), being urban resident AOR: 1.37, 95% CI (0.54, 3.49), perceived good health status AOR: 3.97, 95% CI (1.38, 11.38), having higher income AOR: 4.44, 95% CI (1.32, 14.86), Daily internet use AOR: 2.96, 95% CI (1.08, 6.76), having good knowledge about the availability and importance of online resources AOR: 3.12, 95% CI (1.61, 5.3), having positive attitude toward online resources AOR: 2.94, 95% CI (1.07, 3.52) and higher level of computer literacy AOR: 3.81, 95% CI (2.19, 6.61) were the predictors positively associated with higher eHealth literacy level.

**Conclusion:**

Besides the mounting indication of efficacy, the present data confirm that internet use and eHealth literacy level of chronic patients in this setting is relatively low which clearly implicate that there is a need to fill the skill gap in eHealth literacy among chronic patients which might help them in finding and evaluating relevant online sources for their health-related decisions.

## Background

Noncommunicable diseases (NCDs) are “also known as chronic diseases, which tend to be of extended period resulted from a combination of genetic, physiological, environmental and behavioral factors” [1]. According to the world health organization’s (WHO) report in 2018, non-communicable diseases (NCDs) are accounted for the deaths of 41 million people each year, equivalent to 71% of all deaths globally [1]. In Ethiopia, 39% of all deaths are estimated to be due to Non-communicable diseases [2]. Worldwide, Numerous activities and initiatives are undertaking to alleviate this huge number of incidence and death by one third as indicated in United Nations sustainable development goals (SDG) by 2030 [3]. Literatures depicted that patient empowerment and education focused on self-management and health promotion is very important [4, 5]. Enabling patients to ask about their condition, to follow medication instructions, to improve their adherence and to enhance their engagement in the healthcare process is not an easy task. But, helping patients to help themselves is a key step in improving the overall health status and even in reducing the patient load in hospitals specially in low-income countries [6–8].

The development in internet access and the improvements in performance due to the new technologies makes the internet to be the focus of many new healthcare improvements [9, 10]. Systematic reviews conducted in different chronic disease cases have steadily stated that eHealth interventions for people with chronic diseases have considerable impact on enhancing self-management and patient engagement for their healthcare [11–14]. Despite of the limited number of studies from the perspective of chronic patients in middle and low-income countries, eHealth literacy is pointed in several literatures as one of the major skill required to be acquired by patients with chronic diseases if eHealth interventions are tailored to improve patient’s self-management and facilitating patient’s engagement in the care service [11, 15, 16]. In addition to the low internet penetration in Ethiopia (15%), the gap in skill to find and evaluate online resources is another challenge. E-Health literacy is defined as “one’s ability to seek, find, understand, and appraise health information from electronic sources and apply the knowledge gained for addressing or solving a health problem.” [17]. As the concept of eHealth literacy is a function of different set of skills, it is highly affected by multidimensional factors including sociocultural, economical and behavioral factors [18]. According to the Lily model, eHealth literacy is a congregate set of six basic skills (traditional literacy, health literacy, information literacy, scientific literacy, media literacy and computer literacy) [19]. The details of each components are available in the attached table as Additional file.

Although it is not mandatory to have a mastery in all the six literacy skills, the gap in any of these could be a constraint to benefit from eHealth merits. As a result, one has to have at least a moderate level of skills in all the literacies to make an effective use of the outrageous eHealth resources. To explore the eHealth literacy level and its associated factors in a population with chronic disease is more urgent and important now than ever. The lesson from COVID-19 thought the world that eHealth is not optional and luxurious approach to healthcare rather it is necessary, safer and effective way of providing healthcare service for both patients with underlying conditions and others during such times. The escalating number of internet users, information needs and smartphone penetration in African countries are the most important driving reasons ringing the relevance of assessing eHealth literacy & factors thereof among patients to harness eHealth merits. Given the gap in literatures regarding chronic patients’ eHealth literacy skill in Africa, conducting this study will contribute to the scientific knowledge in addressing the research question in this region. Hence, the aim of this study was to assess eHealth literacy level and associated factors among internet user chronic patients in University of Gondar comprehensive specialized hospital, North West Ethiopia.

## Methods

### Study design and setting

An institutional based cross-sectional study was conducted to assess eHealth literacy skill and associated factor among internet user chronic patients in University of Gondar comprehensive specialized hospital (UOGCSH). Gondar is placed 725-km driving distance North-west from the capital Addis Ababa. The UOGCS hospital is the largest specialized teaching hospital in the Amhara region serving more than five million peoples annually. It provides a specialized care and follow up service for different chronic cases in different days of a week. According to the hospital’s statistics, cardiovascular diseases like hypertension, chronic respiratory diseases like asthma, epilepsy and type2 diabetes are the most common chronic disease morbidities in the hospital at the time when this study was conducted (2019).

### Sample size and procedure

The sample size for this study was calculated using single population proportion formula [20] with 95% confidence level, proportion of eHealth literacy 50% [21] since there were no previous study done in this study population in Ethiopia and relative precision to be 5%. With 10% none-response rate, the total sample size calculated was 423. The participants for this study was selected using a stratified *random sampling* method with *proportional* allocation technique. Stratified sampling is a method of sampling applied for a population with sub-sets of known size where the sub-sets make up different proportions of the whole [22]. Thus, it is appropriate to use stratified random sampling to have a representative sample of the population under study. Participants were approached randomly and the stratification was based on the most prevalent chronic diseases in University of Gondar Comprehensive Specialized Hospital (cardio vascular disease, chronic respiratory disease, diabetics, epilepsy and other chronic diseases were categorized as “other chronic diseases”) hoping for an eHealth literacy difference among patients with different chronic cases [2].

### Data collection instrument and procedure

Data was collected by using interviewer administered questionnaire from February to May 2019. The questionnaire requires 10–15 min to complete and had three parts. The first section contained questions about participant’s socio demographic characteristics (10 items), the second part contained items related with internet use (6 items), knowledge about the availability and importance of online resources (5 items), attitude of participant to use online health resources (4), computer literacy (5 items) and the third section contained items related to eHealth literacy skill (8 items). Except for the first part (sociodemographic related items), all items were measured in 5-point Likert scale ranging from strongly agree (5) to strongly disagree (1). A validated tool was used to determine eHealth literacy skill which was developed by Norman and Skinner [23, 24]. The eHEALS (eHealth literacy scale) is an eight-item scale used to asses self-reported capability of eHealth consumers to find, appraise, and use health related information from the internet to solve health problems [23]. According to Norman and Skinner, the eHealth Literacy Scale (eHEALS) is a promising tool to evaluate users’ ease and skills to use the internet in order to obtain health related information [19, 23]. Apart from the measurement tool coined by Norman and Skinner, researchers have also suggested and used a modified versions of the original eHEALS tailored to their context and variable of interest [25, 26]. Moreover, researchers have also developed a novel measurement tools to assess eHealth Literacy [27–29]. Yet the widely used and constantly validated measurement scale for consumers eHealth Literacy is the original eHEALS proposed by Norman and Skinner. The scale had been used in various studies in different populations exhibiting considerable reliability and validity of items [26, 30–36]. As the eHEALS aims to asses a wide-ranging summary of literacy skills, it is a potential instrument to evaluate the comprehensive literacy skill of eHealth consumers.

The items were translated to Amharic to avoid language barriers and the reliability of the translated items were assessed for its internal consistency by calculating Cronbach alpha coefficient among 30 chronic patients in other hospital (Bahirdar Felegehiwot Referral Hospital) [37]. Data collectors with minimum of diploma in Health Information Technician (HIT) were recruited and trained for data handling and related issues. Participants were approached for consent and data collection while they are on the queue waiting for their turn to see their doctors.

### Data quality control & analysis methods

A one-day training was delivered to data collectors by the investigator in regard with data handling and participants approach. Continuous and active supervision was done during the data collection and after data collection was completed, it was checked for completeness and accuracy. Finally, the data was entered using statistical package for social science (SPSS) software version 20 for further analysis.

Descriptive analysis was performed to summarize participant’s socio-demographic characteristics. The extent of internet use, knowledge and attitude were described using frequency and percentages. Before running the logistic regression model, assumptions were checked for outliers, multicollinearity and independent error terms. Multicollinearity was tested by running a false linier regression iterating the independent variables as a dependent variable and the result showed all the variance inflation factor (VIF) value less than three and tolerance greater than 0.7 which demonstrated the absence of multicollinearity [38]. The data was also checked for outliers by box plot and no outshining outlier effect was observed. The models’ goodness of fit was also checked using omnibus tests of model coefficients for overall (global) fitness of the model and Hosmer and Lemeshow test for fitness of the data to the model. Consequently, the omnibus test result was significant with *p*-value < 0.05 and the Hosmer and Lemeshow test shows a good model fit with *p*-value = 0.608, which signifies the goodness of the model [39]. A bivariate logistic regression was performed for each independent variable and a *p*-value < 0.2 [40] was included in the final model. A multivariable logistic regression was fitted to identify significant factors (*P*-value< 0.05) associated with eHealth literacy skill. The strength of association was explained in terms of odds ratio (OR) and a 95% confidence interval (CI).

## Result

In total, 423 study subjects were approached and included in the study from March 02 to May 17, 2019. The response rate was 95.3%. The majority of respondents 268 (66.3%) were males and the mean age participants was 35.58 ± 14.8 years. A large number of respondents were diploma holders and above 208 (51.5%). Most of the study participants were urban residents352 (87.1%). About 150 (37.1%) of the respondents were governmental employees. See (Table 1) for detail descriptive statistics.

**Table 1:** Descriptive statistics of the participants socio-demographic and other variables

Variable	n	%	Variables	n	%
Age			**Occupation type**		
< 29	203	50%	Governmental	150	37.1%
30–49	110	27.4%	Private	115	28.5%
> 50	91	22.6%	unemployed	139	34.4%
Gender			**Residence**		
Male	268	66.3%	Rural	52	12.9%
Female	136	33.7%	Urban	352	87.1%
Marital Status			**Self-Reported Health Status**		
Single	158	39.1%	Very bad	2	.5%
Married	213	52.7%	bad	39	9.7%
Divorced	20	5.0%	Not bad	105	26.0%
Widowed	13	3.2%	good	225	55.7%
Educational status			Very good	33	8.2%
No formal Education	15	3.7%	**Follow-up in years**		
Primary education	60	14.9%	< 1	105	26.0%
Secondary education	121	30.0%	1–3	136	33.7%
Diploma and above	208	51.5%	3–5	49	12.1%
Type of Chronic Disease			> 5	114	28.2%
CVD	143	35.4%	**Frequency of internet use**		
CRD	24	5.9%	Daily	190	47.0%
Diabetes	141	34.9%	Several days a week	163	40.3%
Epilepsy	52	12.9%	One day a week	28	7.0%
Others	44	10.9%	Less than one day a week	23	5.7%
Monthly Income*					
< 800	118	29.2%			
800–1500	62	15.3%			
1500–3500	99	24.5%			
3500–5500	65	16.1%			
> 5500	60	14.9%			

* The monthly income currency is in Ethiopian Birr (ETB)

### Internet use

The result indicated a 47.0% daily internet use of chronic patients with 81.2% usage for browsing social media. About 95.8% of the participants reported that their major means of internet use was smartphone. Concurrently, 83.4% of those internet user chronic patients confirmed that their major sources of health information were health professionals followed by television broadcasts which was 30.0%.

### eHealth literacy level

The mean eHealth literacy score was 24.6 with a standard deviation of 6.4. Scores less than the median value was labeled as low eHealth literacy and scores greater than & equal to the median value was labeled as high eHealth literacy level. Associated factors with *p*-value less than 0.25 from the bivariate analysis were included in the final multivariable logistic regression model to control the effect of confounding. Accordingly, the statistically insignificant results were Gender, marital status, type of chronic disease and number of medications. From the socio-demographic variables, age, educational status and residence were significantly associated with frequency of internet use where younger, educated and peoples from urban residence were more frequent users of internet. On the other hand, only educational status and residence were significantly associated with knowledge of participants about the availability and importance of online health information. Attitude of chronic patients towards using online sources were primarily associated with their educational status, residence and their monthly income. Whereas computer literacy was associated with Gender, marital status, frequency of internet use and knowledge about the availability and importance of online sources.

The multivariable logistic regression model indicated that participants holding diploma and above are more likely to possess high eHealth literacy skill with AOR: 3.48, 95% CI (1.54, 7.87). similarly, being government employee AOR: 1.71, 95% CI (1.11, 2.68), being urban resident AOR: 1.37, 95% CI (0.54, 3.49), perceived good health status AOR: 3.97, 95% CI (1.38, 11.38), having higher monthly income > 5500ETB AOR: 4.44, 95% CI (1.32, 14.86), being daily internet user AOR: 2.96, 95% CI (1.08, 6.76), having good knowledge about the availability and importance of online resources AOR: 3.12, 95% CI (1.61, 5.3), having positive attitude toward using online resources AOR: 2.94, 95% CI (1.07, 3.52) and higher level of computer literacy AOR: 3.81, 95% CI (2.19, 6.61) were the predictors positively associated with higher eHealth literacy level. See Table 2 for detail.

**Table 2:** Factors associated with eHealth literacy among chronic patients in UOGCSH

Independent Variable	eHealth literacy skill		COR (CI)	*P*-value	AOR (CI)	*P*-value
Educational Status	low	high		< 0.001		0.006
Primary education and below	52	23	1			
Secondary education	84	37	0.99 (0.53, 1.86)	0.892	1.71 (0.74, 3.96)	0.214
Diploma and above	80	128	3.62 (2.06, 6.36)	< 0.001	3.48 (1.54, 7.87)	0.003
Occupation Type				0.037		0.019
Governmental job	69	81	1.85 (1.16, 2.95)	0.024	1.73 (1.11, 2.68)	0.006
Privet job	62	53	1.35 (0.82, 2.22)	0.058	1.02 (0.89, 1.67)	0.314
unemployed	85	54	1			
Residence						
urban	175	177	3.77 (1.88, 7.57)	< 0.001	1.37 (0.54, 3.49)	0.09
rural	41	11	1			
Self-reported health status				< 0.001		< 0.001
bad	32	9	1			
Not bad	66	39	2.10 (0.91, 4.86)	0.08	1.36 (0.45, 4.14)	0.058
good	118	140	4.23 (1.94, 9.19)	< 0.001	3.97 (1.38, 11.38)	0.01
Monthly income*				< 0.001		0.003
< 800	70	48	1			
800–1500	40	22	0.80 (0.42, 1.52)	0.12	0.59 (0.22, 1.57)	0.12
1500–3500	68	31	0.67 (0.38, 1.17)	0.15	0.62 (0.24, 1.59)	0.08
3500–5500	28	37	1.93 (1.04, 3.56)	0.036	1.38 (0.52, 3.73)	0.042
> 5500	10	50	7.29 (3.37, 15.78)	< 0.001	4.44 (1.32, 14.86)	0.016
Frequency of internet use				< 0.001		0.06
Daily	88	102	4.78 (2.18, 10.45)	< 0.001	2.96 (1.08, 6.76)	0.042
Several days a week	92	71	3.22 (1.46, 7.10)	0.004	1.81 (0.71, 4.6)	0.086
One day and less than one day in a week	36	15	1			
Knowledge						
Good Knowledge	54	121	5.42 (3.53, 8.32)	< 0.001	3.12 (1.61, 5.3)	< 0.001
Poor knowledge	162	67	1			
Attitude						
Favorable Attitude	49	110	4.81 (3.12, 7.39)	< 0.001	2.94 (1.07, 3.52)	0.029
Unfavorable attitude	167	78	1			
Computer literacy						
High computer literacy	41	128	9.11 (5.76, 14.39)	< 0.001	3.81 (2.19, 6.61)	< 0.001
Low computer literacy	175	60	1			

*Monthly income currency is in Ethiopian Birr

## Discussion

The purpose of this study was to determine the level of eHealth literacy skill and associated factors among internet user chronic patients in University of Gondar Comprehensive Specialized Hospital. The result showed that eHealth literacy skill was relatively low with 188 (46.5%) of the participants reporting high eHealth literacy skill. The result also shows 353 (87.3%) use of daily and several days a week internet use of participants with more than three fourth of them using it for browsing social medias like Facebook. Younger, educated and peoples from urban residence were more frequent users of internet. Educational status, occupation type, residence, self-reported health status, monthly salary, frequency of internet use, knowledge about the availability and importance of online resources, attitude toward using online resources and computer literacy level were statistically significant factors associated with eHealth literacy skill among internet user chronic patients in this study. This study also found that, the majority of respondents 268 (66.3%) were males and there was no significant chi square difference among the groups which is different from a study which revealed that males are superior internet users [41]. This could be due to the fact that the participants in this study are all internet users. The mean age was 35.58 ± 14.8 years and when compared to elders, youngsters were more frequent internet users. This could be due to the internet penetration rate among youngsters which is related with internet using habit [42].

when compared to studies conducted in other countries, the mean eHealth literacy score of chronic patients in this study was relatively lower and this could be due to the less internet penetration and economic berries in low-income countries like Ethiopia [43–45]. The univariate analysis indicated that patient’s educational status, occupation type, permanent residence, self-reported health status, monthly salary, frequency of internet use, knowledge about the availability and importance of online health information resources, attitude towards using online sources and computer literacy level of participants were significantly associated with eHealth literacy skill with 95% confidence interval, (*p*-value< 0.05). After adjustment for the covariates, except for two variables (residence and frequency of internet use) all the other predictor variables were significantly associated with eHealth literacy skill with *p*-value< 0.05. See Table 2 for detail. Patients with diploma and above education level were 3.48 (CI: 1.54, 7.87) times more likely to have high eHealth literacy skill compared with those with primary and below education level (*p*-value< 0.05). This finding is similar with other studies in different populations which underlined the need for capacity building and continuous educational support for chronic patients with lower educational status [15, 46, 47]. Participants who works in governmental organizations were more likely to possess high eHealth literacy when compared to unemployed chronic patients with AOR: 1.73, 95%CI: (1.11, 2.68). This study also indicated that chronic patients living in urban setting are COR: 3.77 (95%CI: (1.88, 7.57) times more likely to possess high eHealth literacy skill compared to patients residing on rural setting. The finding seems obvious in most cases [48, 49] but it is different in developed countries in which majority of rural patients have computer and internet connection in their home preferring internet for health information [50–52]. Income was the other significant factor associated with eHealth literacy skill among chronic patients in which patients those who have a monthly income > 5500 ETB are more likely to have higher eHealth literacy skill compared with those who earn monthly income < 800 ETB with AOR:4.44, (95%CI, 1.32, 14.86), *P*-value< 0.05. Higher income is highly associated with higher eHealth literacy skill in different studies [53, 54]. In this study, patients who perceived their health status as good were 3.97 times more likely to have higher eHealth literacy skill with 95% CI: (1.38, 11.38) and *p*-value< 0.05 compared to those who perceive their health status as bad. This finding is similar with other studies done elsewhere which argues the more likely possession of eHealth literacy skill by patients who perceive their health status as good [30, 55, 56]. This could be due to the fact that peoples with perceived good health condition are more likely to be looking for possible medical treatment and preventive actions before their health get worse. Frequency of internet use was also the predictive variable with *p*-value< 0.05 where chronic patients those who use internet on a daily basis are almost 3 (AOR: 2.96) times more likely to have high eHealth literacy skill when compared to those who use internet 1 day and less than 1 day a week with 95%CI: (1.08, 6.76). This finding is similar with different studies confirming that frequent internet users possess higher eHealth literacy skill [15, 30, 57, 58]. In addition, higher computer literacy was also highly associated with higher eHealth literacy skill which is similar with studies done elsewhere [59–61]. Knowledge regarding the availability and importance of health information and attitude toward using online resources were significant factors associated with eHealth literacy skill where patients with good knowledge and favorable attitude toward online resources are more likely to possess high eHealth literacy skill with AOR: 3.12, 95% CI: (1.61, 5.3) and AOR: 2.94, 95%CI: (1.07, 3.52) respectively. This finding was also in line with other studies [62–65].

## Conclusion

This study found that educational status, perceived health status, income and frequency of internet use have significant impact on consumers eHealth literacy skill confirming with several studies. It also suggested other factors like knowledge about availability and importance of online sources, attitude and computer literacy are important variables associated with eHealth literacy level in this population. Internet use and eHealth literacy level of chronic patients in this setting is relatively low. This clearly implicate the need to fill the skill gap in eHealth literacy among chronic patients which may help them in building their capacity to find and evaluate relevant online sources for their health-related decisions.

### Limitation of the study

As this is the first study to investigate eHealth literacy and associated factors among chronic patients in resource limited settings, there were limitations identified for further investigators. This limitation of this study is that more than half of the study participants were from urban resident setting and all participants were selected after confirming their previous internet exposure. Those with previous internet exposure were interviewed further and those who doesn’t know about the internet were excluded. This could have caused a selection bias where urban residing and internet user participants are more likely to possess high eHealth literacy. The other limitation of this study was the small sample size & it was conducted in a single institution in Northern Ethiopia. Therefore, the results may not be generalizable to all chronic patients and future researchers should consider more broader samples from different clusters of populations in the country to ensure generalizability.

### Practical implications

Considering the importance of chronic patients’ ability to find and evaluate health information for their health management decisions, it is highly important for the Ethiopian Federal ministry of health and other responsible bodies to consider methodical approach to improve the eHealth literacy skill of internet user chronic patients through short trainings and education. Hospitals should also consider facilitating health care providers advisory of online sources for their patients. It is also important for future researchers to consider exploring other relevant factors associated with eHealth literacy skill of chronic patients.

## Supplementary information

**Additional file 1.**

## Data Availability

All data generated or analyzed during this study are included in this published article.

## Abbreviations

eHEALS: eHealth Literacy Scale
AOR: Adjusted Odds Ratio
CI: Confidence Interval
NCDs: Noncommunicable Diseases
WHO: World Health Organization
eHealth: Electronic Health
UOGCSH: University of Gondar Comprehensive Specialized Hospital
SPSS: Statistical Package for Social Science
VIF: Variance Inflation Factor
OR: Odds Ratio
CVD: Cardiovascular Diseases
CRD: Chronic Respiratory Diseases
COR: Crude Odds Ratio
