# Supplementary material for: E-health literacy and associated factors among chronic patients in a low-income country: a cross-sectional survey
Source: BMC Med Inform Decis Mak. 2020 Aug 6;20:181. doi: 10.1186/s12911-020-01202-1 (PMC7407428; doi:10.1186/s12911-020-01202-1)
Supplement: Supplementary file 1 — Additional file 1. [file 12911_2020_1202_MOESM1_ESM.docx]

**Components of eHealth literacy**

| **Components** | **Description** |
| --- | --- |
| Traditional Literacy | Traditional literacy is the most common understanding of the public to designate who is literate and who is not. It is mainly based on individual’s ability to interact with textual based communications/information which includes reading the text, conceptualizing and responding with written words if necessary [1, 2]. When it comes to the internet world, it becomes as simple as tweeting and retweeting. It seems easy for most peoples who are reading this but there are millions of people out there in Africa who cannot read or write a statement [3]. Thus, traditional literacy is an important and basic literacy skill that contributes much in understanding eHealth literacy because, if one is not “traditionally literate” or unable to read and write, it will be difficult to think about “eHealth” either. As a result, the incorporation of traditional literacy in assessing eHealth literacy skill will widen the perspectives of understanding the concept of eHealth literacy skill in detail. |
| Health literacy | The term “Health literacy” is defined as individual’s performance to comply with medical instructions. In other word, it means the ability to apply the traditional literacy skill set to health related issues like following medical instructions and understanding appointment cards [4]. Studies conducted among chronic patients indicated that lower health literacy is significantly associated with lower disease -related quality of life [5, 6]. To read, understand and use health related information is another important concept to be integrated to understand eHealth literacy. |
| Information literacy | Information literacy is an individual’s knowledge regarding how information is organized, searched and used in an appropriate manner [7]. In this era of cyber world, information literacy is not just an additional skill for an individual, rather it is a mandatory and competitive skill to survive best. After the internet boom, a huge amount of information has been availed to be accessed from anywhere in the world. To know where and how to find an information from the internet certainly require an additional knowledge and skill. This skill is an important dimension to understand individual’s eHealth literacy skill as well. |
| Scientific literacy | As discussed by [*Rüdiger*](https://onlinelibrary.wiley.com/action/doSearch?ContribAuthorStored=Laugksch%2C+R%C3%BCdiger+C) [8], the concept of scientific literacy is a broader and important component of eHealth literacy focusing on understanding the nature, purpose, approaches, needs and limitations of creating knowledge systematically. An individual who is scientifically literate is expected to understand the findings from scientific studies, to apply scientific theories/principles appropriately and accurately, use scientific ways of problem solving and decision making. Scientific literacy is also a relatively complex and educational experience-based literacy which helps in understanding eHealth literacy skill from different perspective. |
| Media literacy | Media literacy is defined as the ability to get, evaluate, share and create a concept from different media contexts including social media, broadcast medias and many more [9]. It is an essential set of skill that helps people to understand the responsible sharing of information, social interaction and wise use of medias. It is considered as a combination of both cognitive and critical thinking progression applied when using media and information from media [10]. Although the wide range of smart phone access and use highly increased the number of internet users the past decades in low- income countries, it was basically driven by social media networking [11]. Thus, incorporating media literacy as a component of eHealth literacy is very important. |
| Computer literacy | Computer literacy is defined as the ability to utilize computers in order to perform a certain task or to solve problems [12]. By its nature, computer literacy is a result of several congregating and evolving concept through time and advancement in technology. *Allan Martin* argues the need to maintain up to date level of digital skill. He asserted that computer literacy is not just a threshold one could achieve through certification and diploma at a time rather, it is a temporary and context based concept referring only to the current level of performance [13]. As a result, assessment of the level of computer literacy should be conducted in parallel with the change in the digital environment. Combining these major literacy skills offers a comprehensive and foundational understanding of the concept eHealth literacy. |

1. Gee JP: **What is literacy**. *Negotiating academic literacies: Teaching and learning across languages and cultures* 1998:51-59.

2. Norman CD, Skinner HA: **eHealth Literacy: Essential Skills for Consumer Health in a Networked World**. *J Med Internet Res* 2006, **8**(2):e9.

3. **Millions in Africa still cannot read or write: report. [ONLINE] Available at: https://**[**www.enca.com/world-literacy-day-highlights-literacy-rates-africa**](http://www.enca.com/world-literacy-day-highlights-literacy-rates-africa)**. [Accessed 5 July 2019].**

4. Nutbeam D: **Health literacy as a public health goal: a challenge for contemporary health education and communication strategies into the 21st century**. *Health promotion international* 2000, **15**(3):259-267.

5. Apter AJ, Wang X, Bogen D, Bennett IM, Jennings RM, Garcia L, Sharpe T, Frazier C, Ten Have T: **Linking numeracy and asthma-related quality of life**. *Patient Education and Counseling* 2009, **75**(3):386-391.

6. Shone LP, Conn KM, Sanders L, Halterman JS: **The role of parent health literacy among urban children with persistent asthma**. *Patient Education and Counseling* 2009, **75**(3):368-375.

7. **Opportunities to Develop Information Literacy. [ONLINE] Available at:** [**http://www.ala.org/acrl/publications/whitepapers/presidential**](http://www.ala.org/acrl/publications/whitepapers/presidential)**. [Accessed 5 July 2019].**

8. Laugksch RC: **Scientific literacy: A conceptual overview**. *Science Education* 2000, **84**(1):71-94.

9. **Media Literacy**. In: *The International Encyclopedia of Communication.*

10. Potter WJ: **Theory of media literacy: A cognitive approach**: Sage Publications; 2004.

11. Stork C, Calandro E, Gillwald A: **Internet going mobile: internet access and use in 11 African countries**. *info* 2013, **15**(5):34-51.

12. Childers S: **Computer literacy: Necessity or buzzword?** 2003.

13. Martin A: **A european framework for digital literacy**. *Nordic Journal of Digital Literacy* 2006, **1**(2):151-161.
